# Preferences of healthcare providers regarding future follow-up care for breast, prostate, and colorectal cancer: A discrete choice experiment

**DOI:** 10.1007/s00520-026-10538-9

**Published:** 2026-03-15

**Authors:** Geertje B. Liemburg, Mariken E. Stegmann, Jako S. Burgers, Annette J. Berendsen, Marjolein Y. Berger, Carolien P. Schröder, Joke C. Korevaar, Daan Brandenbarg

**Affiliations:** 1 Department of Primary and Long-Term Care, University Medical Center Groningen, University of Groningen, P.O. Box 196, 9700 AD Groningen, The Netherlands; 2 Department of Family Medicine, Care and Public Health Research Institute (CAPHRI), Maastricht University, Maastricht, The Netherlands; 3 Department of Medical Oncology, Netherlands Cancer Institute, Amsterdam, The Netherlands; 4 Netherlands Institute for Health Services Research (NIVEL), Utrecht, The Netherlands; 5 Faculty of Health, Nutrition and Sport, The Hague University of Applied Sciences, The Hague, The Netherlands

**Keywords:** Cancer survivorship, Discrete choice experiment, Task substitution, Breast cancer, Colorectal cancer, Prostate cancer

## Abstract

**Background:**

With rising cancer survival rates and an increasing number of cancer survivors, the sustainability of secondary care follow-up care is under pressure. Transferring certain follow-up tasks to primary care is suggested as a potential solution, but there is no consensus on the optimal strategy for this.

**Purpose:**

To identify preferences of primary and secondary healthcare providers regarding the transfer of follow-up care for breast, colorectal, and prostate cancer, using a discrete choice experiment (DCE).

**Method:**

A DCE was conducted among 153 primary and secondary healthcare professionals in the Netherlands. Attributes related to patient and follow-up characteristics included: type of cancer, age, time post-treatment, any protocolled care for comorbidities, recurrence risk, and types of check-up protocols. A conditional logit model and latent class analysis were used to assess preferences and identify subgroups.

**Results:**

Healthcare providers favored the transfer of follow-up care to primary care for prostate cancer patients. Across all cancer types, preferences for substitution were greater in patients aged above 75 years, longer post-treatment, a low recurrence risk, and who were already enrolled in protocolized chronic care. Transfer of basic check-up care was preferred, while extensive check-up was negatively valued, especially by GPs.

**Conclusion:**

Future follow-up should focus on patient groups and tasks for which consensus exists that primary care involvement is feasible and appropriate, forming the basis for sustainable, collaborative, and patient-centered models of care.

**Supplementary Information:**

The online version contains supplementary material available at 10.1007/s00520-026-10538-9.

## Introduction

The number of cancer survivors is increasing due to several factors, including advancements in early detection and screening and improved treatment options [1]. By 2050, the number of new cancer cases worldwide is projected to exceed 35 million, representing a 77% increase compared to the estimated 20 million cases in 2022 (basal cell carcinoma excluded) [2, 3]. More than two-thirds (68%) of cancer patients are expected to survive the first 5 years following their diagnosis, with 5-year survival rates of 89%, 67%, and 90% for breast cancer, colorectal cancer, and prostate cancer, respectively, in the Netherlands [4]. Upon completion of cancer treatment, survivors are typically enrolled in secondary care follow-up programs lasting up to 5 years for colorectal cancer and 3 years (with possible extension up to 5 years) for breast cancer, according to recent guidelines [5–9].

Breast, colorectal, and prostate cancer were selected for this study because they are among the most frequently diagnosed cancers and together account for a large proportion of long-term cancer survivors [2–4]. In addition to their high incidence, these cancers are characterized by relatively favorable prognoses and structured, guideline-based follow-up trajectories that primarily focus on recurrence surveillance and the management of late and long-term treatment effects [5–9]. Routine follow-up after curative treatment generally involves limited use of highly specialized diagnostics, rendering follow-up care potentially suitable for redistribution across healthcare settings.

The increasing number of cancer survivors is expected to further strain existing shortages in healthcare resources, including financial and staffing limitations [10]. Maintaining the current level of follow-up in secondary care is unsustainable and poses significant challenges to long-term healthcare delivery. Several alternative models for this care have been proposed, such as shifting follow-up care to primary care, but there is no consensus yet on the optimal strategy for oncological follow-up or on the appropriate role of primary care in this context [11]. Further exploration is required to address this issue, enabling future implementation of a supported follow-up strategy [12, 13].

Healthcare providers should be involved in decisions regarding the allocation of follow-up care to maintain high quality of care. Without their guidance, there is a risk that new strategies do not achieve optimal outcomes. Qualitative research involving patients, specialists, and GPs has provided valuable insights into the barriers, facilitators, requirements, and feasibility of primary care involvement in cancer follow-up care [12, 14–16]. The aim of this study is to further quantify these findings by identifying primary and secondary healthcare providers’ preferences regarding care substitution in cancer patients using a discrete choice experiment (DCE). With this, we aim to provide building blocks for the development of sustainable follow-up care.

## Material and methods

### Research methodology

In the current study, we assessed the preferences of primary and secondary healthcare professionals regarding which care and for which patient groups primary care providers could take responsibility in the follow-up care of breast, colorectal, and prostate cancer, three of the most common cancer types.

We performed a cross-sectional survey using a discrete choice experiment (DCE). Respondents were presented with a series of tasks in which they had to choose between two hypothetical scenarios. Each scenario was described using a set of attributes that varied across different levels. For each pair, they indicated which scenario they preferred for substitution of care, specifically, the one they considered most appropriate for general practice to take responsibility for. The series of choices reveal the utility respondents assign to those attributes. Utility is a subjective measure indicating the value a participant places on a specific attribute level; higher utility scores reflect stronger preferences. The method is based on Lancaster’s theory of value and McFadden’s Random Utility Theory [17–20].

### Study participants

Primary care providers (GPs and general practice nurses) and secondary care providers currently involved in follow-up care for breast, colorectal, or prostate cancer (medical specialists, nurse practitioners, and oncology nurses) were recruited for this study. Participants were recruited via Netherlands Institute for Health Services Research (NIVEL), the Academic General Practitioner Development Network (AHON), social media platform LinkedIn, and emails to hospitals throughout the Netherlands. Snowball sampling was used by asking participants to share the DCE link with colleagues. Participants were required to understand Dutch. All participants gave informed consent before participation in the study. Among all participants, 15 gift cards worth €75 each were raffled. The data collection took place from 1 April to 1 September 2022.

### The discrete choice experiment

The design of the DCE followed the best-practice recommendations outlined in the checklist by Bridges et al. to ensure methodological rigor and transparency [18]. The development of the DCE, including attribute selection, pilot testing, and final design, is described below.

#### DCE development

The development of the DCE followed a systematic, multi-step process to ensure content validity and respondent comprehension. An initial list of 12 potential attributes was identified based on prior research, including a systematic literature review, qualitative interviews and focus groups with GPs, secondary care providers, and patients, as well as expert input from our research group [14–16, 21, 22].

These potential attributes were reduced to six final attributes through consensus discussions within the research team, which comprised healthcare professionals from both primary and secondary care with research experience, alongside epidemiologists and a methodologist. Two think-aloud sessions were conducted (one oncologist and one GP) to assess comprehension and decision-making processes. Participants were asked to verbalize their thoughts while completing the choice tasks, and observations were documented. Feedback from these sessions indicated that the scenarios contained excessive detail, especially regarding recurrence risks. This feedback led to simplification of recurrence risk in low/medium/high, to improve clarity and reduce cognitive burden. A subsequent pilot study with 26 respondents was used to estimate Bayesian priors, which were then incorporated into the final experimental design to optimize it as a Bayesian D-efficient design.

#### Attributes and levels

Following the development process described above, the final DCE included six attributes consisting of two or three levels (Table 1). The first three attributes reflect patient and clinical characteristics: type of cancer, age, and time since treatment completion. The fourth captures whether the patient already receives protocolled care for chronic conditions, such as diabetes and cardiovascular risk management, in general practice. The fifth concerns recurrence risk. The final attribute describes the type of follow-up, ranging from a basic check-up to an extensive consultation, including interpretation of complex imaging. Attribute definitions presented to respondents are shown in Supplement 1.

**Table 1:** Attributes and levels included in the DCE

Attributes	Attribute levels	Description
1. Type of cancer	- Breast cancer - Colorectal cancer - Prostate cancer	The type of cancer for which follow-up needs to be performed
2. Age	- < 60 years of age - 60–75 years of age - > 75 years of age	Age range of patient needing follow-up
3. Time since completion of treatment	- 1 years - 2–3 years - 4–5 years	Time since completion of cancer treatment (except anti-hormonal treatment) after which primary care could take responsibility for follow-up
4. Protocolled care due to comorbidity	- No - Yes	Whether the patient already receives regular protocolled care in the GP practice (e.g. for diabetes mellitus or COPD)
5. Recurrence risk	- Low - Medium - High	Recurrence risk (within 5 years after treatment)
6. Type of follow-up protocols	- Basic check-up - Extensive check-up	Protocols according to the applicable guidelines for each tumor type **Basic check-up:** History taking, physical examination, blood tests, and ordering and discussing simple imaging (such as mammography). Other tasks are performed in hospitals **Extensive check-up:** Performing the basic check-up and ordering and discussing complex imaging (including CT-scan, MRI, and PET-scan). The GP can easily consult the hospital for questions

#### Design

**Fig. 1:**
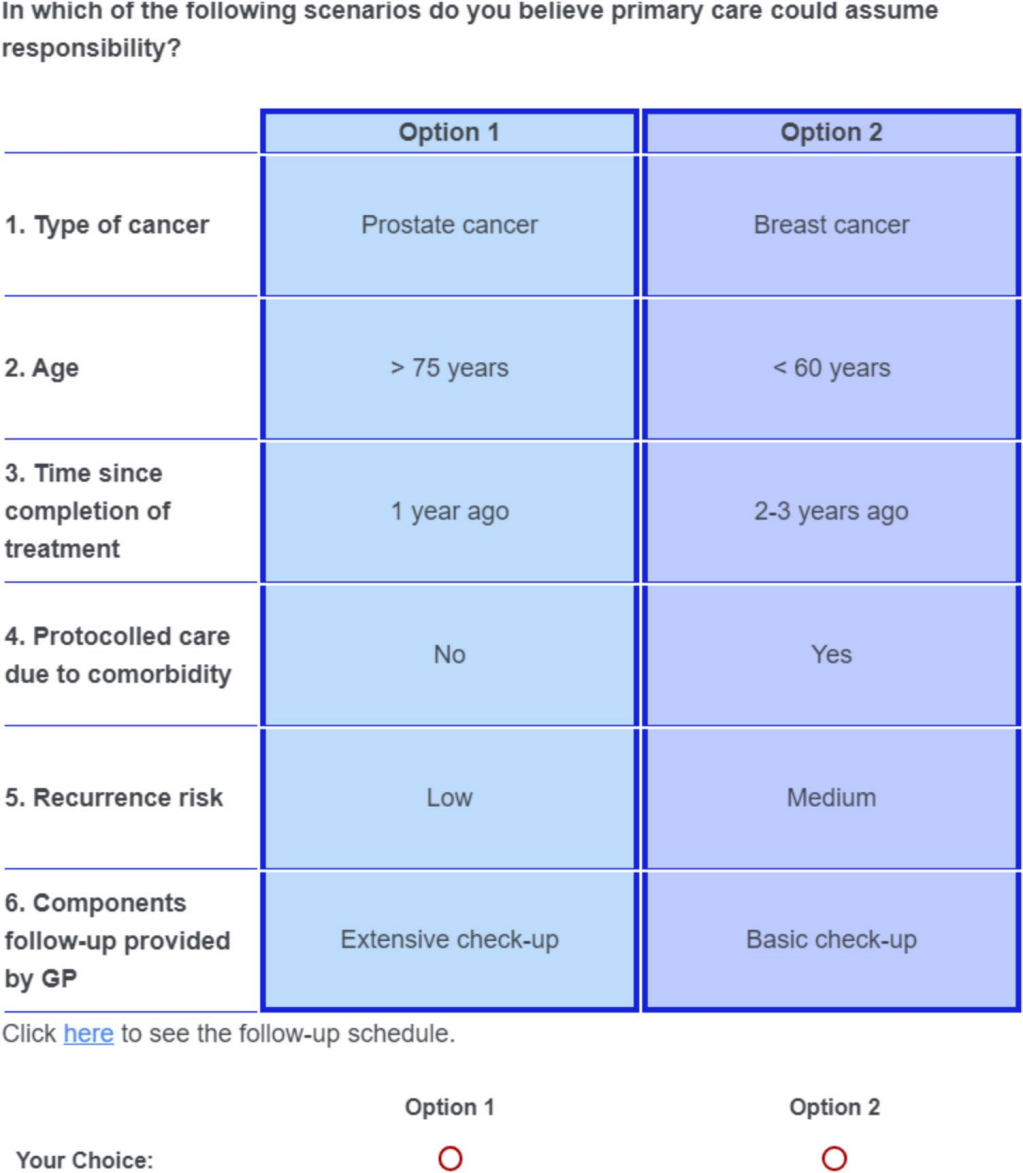
Example of a scenario. Respondents were asked to choose the option they preferred for substitution of care

We employed a fractional factorial design, since a full factorial design with 256 scenarios (4^3^ × 2^2^) was deemed unfeasible due to participant burden. The final design consisted of 18 choices, optimized to maximize information on main effects and selected interactions (D-efficiency) for a conditional logit model, using Ngene 1.3 (ChoiceMetrix) [23]. The questionnaire was programmed in Qualtrics (Qualtrics, Provo, UT) and included one exercise to familiarize respondents with the scenarios. Demographic data (gender, age), specialization, work experience, and a general question on the desirability of care substitution (5-point Likert scale) were also collected. An example scenario is shown in Fig. 1.

Based on established guidelines for sample size in discrete choice experiments, a minimum of approximately 84 respondents is required to estimate main effects reliably, according to the rule-of-thumb formula proposed by Johnson and Orme, assuming two alternatives per task, 18 scenarios per respondent, and a maximum of three levels per attribute [24, 25].

### Statistical analysis

Descriptive statistics were performed for demographics of the included respondents. The variable ‘desirability of substitution’ was dichotomized for analysis of the interactions and latent class analysis into: opponents (very undesirable and undesirable) and supporters (neutral to very desirable). A conditional logit model was fitted to assess the main effect and determine the relative importance of the various attributes. A positive beta (regression) coefficient indicates that an increase in the attribute level is associated with a higher likelihood of choosing that option, whereas a negative beta indicates a decreased likelihood of selection, reflecting participants’ preferences. Specific interactions for type of healthcare provider (primary vs. secondary care) and desirability of substitution between attributes were analyzed by using Wald chi-square tests. To uncover underlying subgroups with similar response patterns in the discrete choice experiment, we conducted a latent class analysis. The number of classes was determined by the lowest consistent Akaike information criterion (CAIC). The characteristics of the identified subgroups were analyzed descriptively. STATA version 15 (College Station, Texas, USA) was used to analyze all data.

## Results

### Respondents

A total of 153 respondents completed the online DCE survey, of whom 115 (75%) were primary care healthcare providers (Table 2). The median age of the respondents was 47 (29–70) years, and 60% were female (*n* = 91). Opinions on the desirability of substitution varied, with 65% of participants in the supportive group (*n* = 98). The median time to complete the survey was 10 min.

**Table 2:** Participants’ characteristics

	**Total** ***n *= 153**	**Primary care** ***n *= 115**	**Secondary care** ***n *= 38**
**Age (years), median (range)**	47 (29–70)	46 (29–70)	48 (29–69)
**Gender (female),** ***n*** **(%)**	91 (60)	68 (59)	23 (61)
**Health care provider,** ***n*** **(%)**			
Primary care	115 (75)		
Secondary care	38 (25)		
**Job title**			
GP		100 (65)	
GP-based nurse		15 (10)	
Hospital specialist			27 (18)
Nurse practitioner			11 (7)
**Years of experience, median (range)**	13 (39)	13 (39)	12 (34)
**Place of occupation**			
Urban GP-practice		50 (33)	
Suburban GP-practice		65 (42)	
Academic hospital			16 (10)
Teaching hospital			11 (8)
General hospital			11 (8)
**Type of cancer follow-up,** ***n*** **(%)**			
Colorectal			15 (36)
Breast			17 (40)
Prostate			10 (24)
**Desirability of substitution,** ***n*** (%)*			
Very undesirable	14 (9)	14 (12)	0
Undesirable	39 (26)	32 (28)	7 (18)
Neutral	54 (36)	43 (37)	11 (29)
Desirable	38 (25)	23 (20)	15 (39)
Very desirable	6 (4)	3 (3)	3 (8)
**Groups**			
Opponents	53 (35)	46 (40)	7 (20)
Supporters	98 (65)	69 (60)	29 (80)

*Data of 2 respondents from secondary care is missing

### Conditional logit model estimation

All attributes had levels that showed significant utility for follow-up care in general practice (Table 3), indicating that each attribute included at least one level that meaningfully influenced respondents’ preferences. The following section presents the results per attribute, highlighting which levels were most influential in shaping preferences. For cancer type, a significantly positive utility was found for substitution of prostate cancer care (0.24, 95% CI [0.08–0.40], *p* < 0.01), whereas a non-significant negative utility was observed for colorectal cancer (−0.10, 95% CI [−0.22–0.02], *p* = 0.11). This indicates that substitution of follow-up care to general practice was most preferred for prostate cancer, least for colorectal cancer, with breast cancer serving as the reference. For patient age, significant positive utility values were found for ages 60–75 (0.47, 95% CI [0.34–0.60], *p* < 0.01) and above 75 years (0.95, 95% CI [0.75–1.16], *p* < 0.01), indicating that follow-up in general practice was increasingly preferred with older age. For time since treatment, significant positive utility values were found for 2–3 years (0.25, 95% CI [0.15–0.35], *p* < 0.01) and 4–5 years (0.48, 95% CI [0.36–0.60], *p* < 0.01), indicating that follow-up in general practice was more acceptable as more time had passed since treatment. For protocolled care, a significant positive utility was found (0.13, 95% CI [0.05–0.21], *p* < 0.01), indicating a preference for follow-up in general practice when patients already receive protocolled care for a chronic condition. Respondents reported a significant negative utility for high recurrence risk (−1.26, 95% CI [−1.52 to −1.00], *p* < 0.01) and moderate recurrence risk (−0.53, 95% CI [−0.66 to −0.39], *p* < 0.01), indicating a clear preference against follow-up in general practice for patients with higher recurrence risk. In addition, extensive check-up was associated with significant negative utility (−1.16, 95% CI [−1.37 to −0.96], *p* < 0.01) compared to basic check-up (reference), indicating that respondents perceived extensive check-up as significantly less desirable.

**Table 3:** Results of conditional logit model based on the preferences of healthcare providers regarding oncological follow-up care

Attribute	*β*	95% Confidence interval		*p*
Cancer (breast cancer)	0.00			
Cancer (colorectal cancer)	−0.10	−0.22	0.02	0.11
Cancer (prostate)	0.24	0.08	0.40	< 0.01
Age (< 60 years)	0.00			
Age (60–75 years)	0.47	0.34	0.60	< 0.01
Age (> 75 years)	0.95	0.75	1.16	< 0.01
Time (1 year ago)	0.00			
Time (2–3 years ago)	0.25	0.15	0.35	< 0.01
Time (4–5 years ago)	0.48	0.36	0.60	< 0.01
Protocolled care (no)	0.00			
Protocolled care (yes)	0.13	0.05	0.21	< 0.01
Recurrence risk (low)	0.00			
Recurrence risk (medium)	−0.53	−0.66	−0.39	< 0.01
Recurrence risk (high)	−1.26	−1.52	−1.00	< 0.01
Type follow-up (basic)	0.00			
Type follow-up (extensive)	−1.16	−1.37	−0.96	< 0.01

*The beta coefficient indicates how the utility of choosing an alternative changes relative to the reference attribute: a positive beta means the utility increases, while a negative beta means it decreases

When examining specific interactions between primary and secondary healthcare providers, the primary care group was significantly negative about extensive check-up (−0.49, 95% CI [−0.93 to −0.05], *p* = 0.029), whereas in the secondary care group, extensive check-up demonstrated a positive utility (0.46, 95% CI [0.13 to 0.90], *p* = 0.043). No significant interactions were found between the desirability of substitution and the various attributes.

### Latent class model estimation

Latent class analysis identified three distinct classes, representing 61%, 28%, and 12% of participants, respectively (Table 4). Class 1, the largest group, showed a preference pattern closely aligned with overall conditional logit results, characterized by positive utilities for older age and longer time since treatment, and strong negative utilities for increasing recurrence risk and extensive follow-up. Class 2 was characterized by a pronounced aversion to extensive follow-up tasks, largely independent of patient characteristics, and included a high proportion of GPs (*n* = 35 out of 40). Class 3 was characterized by strong negative utilities for increasing recurrence risk, combined with higher utility for older patient age, particularly for patients over 75 years. This class also had a relatively high proportion of women (93% vs. 50% and 58% in Classes 1 and 2, respectively), younger age, and less work experience (Table 5).

**Table 4:** Results of the latent class analysis with 3 classes [*β* and 95% confidence intervals]

Attribute	Class 1	Class 2	Class 3
Cancer (colorectal cancer)	−0.01 [−0.17–0.16]	−0.79 [−1.42 to −0.17]*	−0.79 [−1.41 to −0.18]*
Cancer (prostate)	0.55 [0.37–0.72]***	−0.56 [−1.18–0.06]	−0.57 [−1.31–0.17]
Age (60–75 years)	0.56 [0.37–0.75]***	0.46 [−0.14–1.06]	0.73 [0.10–1.34]*
Age (> 75 years)	1.22 [0.98–1.46]***	0.55 [−0.29–1.39]	1.97 [1.14–2.80]***
Time (2–3 years ago)	0.38 [0.21–0.56]***	0.26 [−0.32–0.55]	−0.29 [−0.92–0.33]
Time (4–5 years ago)	0.66 [0.48–0.83]***	0.51 [−0.01–1.03]	0.41 [−0.28–1.10]
Protocolled care (yes)	0.16 [0.04–0.28]**	0.12 [−1.03–0.40]	0.39 [−0.07–0.86]
Recurrence risk (medium)	−0.56 [−0.75 to −0.37]***	−0.32 [−1.03–0.40]	−2.07 [−2.85 to −1.29]***
Recurrence risk (high)	−1.25 [−1.57 to −0.93]***	−1.34 [−2.45 to −0.23]*	−4.63 [−5.91 to −3.35]***
Type follow-up (extensive)	−0.87 [−1.07 to −0.68]***	−3.54 [−4.35 to −2.73]***	−0.30 [−0.83–0.23]
Class share	61%	28%	12%

The bold values represent statistically signifi cant results

*p < 0.05; **p < 0.01; ***p < 0.001

*A positive beta means the utility increases, while a negative beta means it decreases

**Table 5:** Characteristics of the participants in the different latent classes

Characteristics	Class 1 (60.9%) *n* = 98	Class 2 (27.7%) *n* = 40	Class 3 (11.5%) *n* = 15
Male (%)	42	50	7
Female (%)	58	50	93
Age (mean)	47.8	46.6	44.3
Experience years (mean)	15.3	16.0	11.9
Primary care (%)	71	87	73
Secondary care (%)	29	13	27
Undesirable substitution (%)	36	43	13
Neutral-desirable substitution (%)	64	57	87

## Discussion

### Summary

This DCE with 153 healthcare professionals demonstrated that, under forced-choice conditions, providers from both primary and secondary care were more likely to prefer primary care involvement for prostate cancer follow-up than for colorectal or breast cancer, and for older patients, those later in follow-up, and those already receiving protocolled care for chronic diseases in primary care. Patients with a high risk of recurrence were consistently deemed unsuitable for transfer of follow-up care to primary care. With regard to the type of follow-up care that could be transferred to primary care, respondents preferred these to be less extensive, with GPs being more against extensive check-up tasks than secondary care providers.

Latent class analysis revealed two distinct subgroups of healthcare professionals with divergent decision patterns. The subgroup that strongly opposed extensive check-up in primary care, regardless of patient characteristics, consisted mainly of GPs. The other subgroup consisted of younger female healthcare professionals who consistently avoided transferring follow-up care to primary care for high-risk patients. They also preferred older patients to be followed in primary care.

### Comparison with literature

The findings of this study align with previous qualitative research on the management of cancer follow-up care in primary care, particularly regarding the preference for primary care follow-up for certain patient groups. Several studies have demonstrated a similarly positive attitude toward primary care involvement in the follow-up of prostate cancer patients [21, 26]. Additionally, a Dutch feasibility pilot study, in which prostate cancer care was transferred to GPs for one year, showed that both GPs and urologists were confident about the ability of GPs to provide this follow-up care [21]. In contrast, a recent study reported that colorectal cancer was perceived as more complex, with GPs expressing more uncertainty about their knowledge of colorectal cancer compared to their confidence in managing prostate cancer follow-up [27]. A factor that may be taken into account is that during colorectal cancer follow-up carcinoembryonic antigen (CEA) is measured. This can be elevated due to various factors that may lead to additional imaging and colonoscopies. In contrast, breast cancer follow-up is regarded by GPs as more straightforward, with confidence in their role increasing when supported by clear protocols and collaboration with specialists [15].

The preference for primary care management of older patients during extended periods post-treatment, as observed in our study, is consistent with earlier studies [28, 29]. This may be because GPs in The Netherlands often have a long-term relationship and medical history with these patients, which allows them to take contextual and comorbidity-related factors into account. In addition, an increasing number of GP practices employ nurse practitioners specialized in elderly care, further supporting this role. In the earlier phases after treatment, cancer-specific side effects may still be prominent, and previous studies indicate that GPs sometimes lack confidence in managing these, particularly with the advent of newer therapies [15, 30, 31]. This may explain the preference among our participants for primary care involvement mainly at longer times after treatment. The preference for patients already receiving protocolled care in primary care may be explained by logistical factors, as follow-up can be more easily integrated into existing structured visits, often involving routine tests conducted by practice nurses [13]. Our finding that patients with a high recurrence risk were not considered suitable for primary care follow-up is consistent with previous studies that have raised concerns about whether primary care has sufficient clinical expertise to manage high-risk cancer survivors [16, 32, 33].

The latent class analysis revealed that healthcare professionals do not form a homogeneous group, but instead apply distinct decision-making logics when considering transfer of follow-up to primary care. These classes differed not only in the strength of preferences, but also in the way patient and task characteristics were weighed against each other. The largest class appeared to apply a clinically conditional delegation heuristic: primary care follow-up was considered acceptable for older patients and those further from treatment, but only when recurrence risk was low and follow-up tasks remained limited [34]. This pattern aligns with principles of risk-stratified follow-up and suggests a cautious but pragmatic approach to task substitution. A second class, consisting predominantly of GPs, was primarily characterized by a strong aversion to extensive follow-up tasks, largely irrespective of patient characteristics. This pattern suggests a boundary- or workload-protective decision heuristic, in which feasibility and task intensity outweigh clinical differentiation [35]. The third, smaller class demonstrated pronounced sensitivity to recurrence risk, strongly opposing transfer of follow-up for patients with even moderate risk, while favoring primary care follow-up for older patients with low risk. This pattern is consistent with a safety-first decision heuristic, in which primary care involvement is considered acceptable only under conditions of minimal clinical uncertainty. This group predominantly consisted of younger female GPs. A possible explanation may relate to gender differences in risk attitudes, as previous research generally indicates that women tend to exhibit more risk-averse behavior than men [36, 37]. Furthermore, younger female physicians may perceive professional risks differently; studies suggest that younger and female doctors report greater sensitivity to professional risk [38].

### Implications

Given the growing strain on healthcare resources, the findings of this study underscore the need for a more differentiated approach to follow-up care. Decisions should be guided by individual patient risk profiles, preferences, and expected clinical benefit, rather than strictly adhering to one-size-fits-all protocols. The results indicate that follow-up for older patients with a low recurrence risk could be organized in general practice, which is consistent with the central role of GPs in the Dutch healthcare system. Shared decision-making between the GP, the medical specialist, and the patient may reduce the intensity of follow-up in secondary care. Recent revisions to breast and colorectal cancer guidelines emphasize risk-stratified follow-up, including the option to refrain from intensive surveillance in selected groups. To implement this in practice, structured programs embedded in routine care could be initiated. For example, follow-up for low-risk prostate cancer patients aged over 75 years and two years post-treatment could be coordinated primarily in primary care, with clear criteria for re-referral. Starting with clearly defined patient groups allows feasibility, outcomes, and resource implications to be evaluated, providing a foundation for potential stepwise expansion to other cancer types or follow-up settings.

GP’s knowledge of patient history, comorbidities, and social circumstances can help ensure follow-up aligns with patient preferences. However, an expanded role for primary care requires explicit support, including clear task delineation, access to specialist consultation, and practical tools to facilitate communication between primary and secondary care. Considering the growing workload and declining GP capacity, any redistribution of follow-up care should be accompanied by a critical reassessment of the overall necessity and intensity of follow-up. Any increased role for primary care must acknowledge existing capacity constraints and is unlikely to be feasible without additional time and resources, potentially requiring dedicated support such as the involvement of nurse practitioners or reallocation of resources from secondary care.

### Strengths and limitations

The use of a DCE, a grounded research method with a solid theoretical foundation to elicit preferences, is a strength of this study. As with all DCEs, responses may be influenced by hypothetical or social desirability bias, and may not fully reflect actual clinical behavior. Through rigorous preparation and pilot testing, including think-aloud sessions, we developed an efficient DCE design that participants could complete in around 10 min at a time and place of their choosing. Furthermore, this is one of the first DCEs on this topic involving healthcare providers, whereas most previous studies focused on patient preferences. A limitation is that fewer secondary care providers participated than primary care providers. However, this may reflect the Dutch practice, as a relatively small group of specialists performs oncological follow-up, while almost all GPs encounter this in their practice. Sufficient power was available to study main parameters, but we lacked power to estimate more latent classes or perform formal subgroup analyses within the latent class analysis. Some attributes, such as “recurrence risk” and “extensive check-up”, may not fully capture all clinical nuances, limiting interpretation of the preferences elicited. Finally, the response rate could not be precisely determined due to the use of snowball sampling and is estimated at approximately 10%, potentially introducing selection bias as participants may have been particularly interested in cancer follow-up. Moreover, the study was conducted within the Dutch healthcare system, which features strong primary-secondary care integration, limiting generalizability to more fragmented systems.

## Conclusion

This study indicates that cancer follow-up care in primary care is not a one-size-fits-all solution. Rather, transfer should begin with patient groups and tasks for which both primary and secondary care providers agree that primary care involvement is feasible and appropriate. Future efforts should therefore focus on developing sustainable follow-up models that build on this consensus, ensure efficient collaboration between primary and secondary care, and support patient-centered care. To further strengthen these models, future research should explicitly incorporate patient preferences, particularly where these may diverge from provider perspectives. In addition, health economic evaluations, including cost-utility analyses, are needed to assess the economic impact of task substitution.

## Supplementary Information

Below is the link to the electronic supplementary material.

ESM1 (DOCX 28.8 KB)

## Data Availability

No datasets were generated or analysed during the current study.
