# Supplementary material for: Preferences of healthcare providers regarding future follow-up care for breast, prostate, and colorectal cancer: A discrete choice experiment
Source: Support Care Cancer. 2026 Mar 15;34(4):325. doi: 10.1007/s00520-026-10538-9 (PMC12988968; doi:10.1007/s00520-026-10538-9)
Supplement: Supplementary file 1 — (DOCX 28.8 KB) [file 520_2026_10538_MOESM1_ESM.docx]

# Supplement 1: Discrete Choice Experiment as Presented to Participants (English Version)

## Introduction to the Supplement

This supplement presents the Discrete Choice Experiment (DCE) as it was presented to study participants. The DCE explored preferences regarding follow-up care for breast, colorectal, and prostate cancer survivors in primary care.

Participants chose between pairs of scenarios, each described by six varying attributes related to patient characteristics, cancer type, recurrence risk, and potential GP follow-up tasks. Even if neither scenario seemed fully suitable, participants were asked to select the most appropriate option for implementation in general practice.

To familiarize participants, an example question illustrating all six attributes was provided. While completing the DCE, participants were asked to assume:

- The patient had successfully completed curative treatment.
- There were no complications or notable events during treatment.
- The GP would play a larger role in follow-up.

The following part provide the English version of the original DCE, including the six attributes and an overview of current hospital-based follow-up practices during the first five years after treatment in the Netherlands.

## Attributes

- **Type of cancer:** Varies between breast, colorectal, and prostate cancer.
- **Patient age:** Divided into three categories: <60 years | 60–75 years | >75 years.
- **Time since completion of treatment:** Refers to the end of the active treatment phase, excluding ongoing hormone therapy.
- **Protocolled care due to comorbidity:** Indicates whether the patient receives protocolled care in general practice for other conditions, such as diabetes or COPD.
- **Recurrence risk:** The likelihood that a patient will be diagnosed with a recurrence within the first five years after initial diagnosis. Recurrence risk is categorized as Low, Medium, or High.
- **Components follow-up provided by GP**: Refers to the tasks the GP would perform during potential future follow-up:
   • Basic care: History taking, physical examination, blood tests, requesting and reviewing basic imaging.
   • Comprehensive care: Basic care plus requesting and reviewing advanced imaging (including CT and MRI scans), with imaging interpreted by a hospital-based specialist.

## Current Follow-up Practices

The content of hospital-based follow-up during the first five years after treatment is summarized below. There may be slight variations between hospitals and patients. The frequency of follow-up depends on the type of cancer. In the first 2–3 years, patients are on average seen 3–4 times per year, and thereafter 1–2 times per year.

| **Cancer type** | **Breast cancer** | **Colorectal cancer** | **Prostate cancer** |
| --- | --- | --- | --- |
| **Basic check-up** | Detecting recurrence, symptoms, side effects, late effects, psychosocial aftercare | | |
| History taking |  |  |  |
| Physical examination | Palpation of both breasts and axillae | Digital rectal examination if indicated | Digital rectal examination if indicated |
| Blood tests | – | CEA | PSA |
| Basic imaging | Annual mammography/ultrasound | – | – |
| **Comprehensive check-up** | MRI scan if indicated | CT scan thorax/abdomen; colonoscopy | PET-CT scan; MRI scan if indicated |
